# How does rapport impact knowledge transfer from older to younger employees? The moderating role of supportive climate

**DOI:** 10.3389/fpsyg.2022.1032143

**Published:** 2022-12-07

**Authors:** Hainan Rui, Hailong Ju

**Affiliations:** Business School, Guilin University of Technology, Guilin, China

**Keywords:** knowledge transfer, rapport, supportive climate, younger employees, older employees

## Abstract

**Introduction:**

Knowledge transfer from older to younger employees plays a key role in lessening knowledge loss and maintaining firms' competitiveness. While the disharmony derived from a salient age difference between younger and older workers hinders such knowledge transfer. This study aims to construct a rapport model to address it.

**Methods:**

Data from 318 respondents in various industries were collected through a questionnaire-based survey to test the proposed model. The research hypotheses were tested using hierarchical multiple regressions.

**Results:**

Our empirical results show that almost all rapport dimensions facilitate such knowledge transfer; The moderating role of supportive climate is strong that it enhances or replaces the effects of rapport dimensions on such knowledge transfer.

**Discussion:**

This study contributes to research on knowledge transfer and rapport by providing a detailed understanding of the relational mechanism of the knowledge transfer from older to younger employees based on a revised model of rapport. It also serves as a reference for firms to leverage rapport-building and a supportive climate to enhance this invaluable knowledge transfer.

## Introduction

How can the severe loss of knowledge caused by the aging and retirement of skilled workforces be mitigated? An important resolution lies in the retention of valuable knowledge from older to younger employees. Given the differences in ways of thinking, attitude, behavior, and value system of the two cohorts (Starks, 2013; Bencsik et al., 2016), there is disharmony within their relationships, obstructing knowledge transfer (Schmidt and Muehlfeld, 2017). Researchers argued that the knowledge of older employees is valuable but mainly tacit, demanding more relational harmony to be transferred (Magni et al., 2018). Additionally, the transfer of such knowledge is not automatical and must be effectively received by younger workers who especially desire to keep harmony in relationships (Zhang et al., 2005). Building harmonious relationships could therefore support the active participation of younger workers in acquiring knowledge from older counterparts, through which firms avoid irrevocable knowledge loss and therefore maintain competitiveness.

Existing knowledge transfer literature on interpersonal relationships between knowledge senders and receivers focuses primarily on the roles of trust and willingness. Concerning trust, it has been identified as an enabler of relationship strength that expedites the transfer of knowledge (Bacon et al., 2020; Vasin et al., 2020; Bettis-Outland et al., 2021). With regard to willingness, researchers discovered that positive interpersonal relationships could increase the willingness of employees to transfer knowledge (Anand et al., 2019; Nguyen et al., 2020). When it comes to knowledge transfer from older to younger employees (KTOYE), the upcoming retirement results in older employees' natural willingness to share knowledge with younger ones. In contrast, the younger cohort desires growth opportunities and is willing to learn through knowledge transfer (Fasbender and Gerpott, 2021). However, older workers focus more on the positive aspects of relationships, making them trust younger workers (Bal et al., 2011). In addition, younger ones spontaneously trust their older colleagues who possess wisdom and experience (Kmieciak, 2021). It can be seen that trust and willingness are already at a high level in KTOYE; however, disharmony exists between the two cohorts, hindering their knowledge transfer. Such disharmony exists especially when knowledge senders and receivers have a pronounced age difference, for which they perceive each other dissimilarly in aspects of values, behaviors, and identity (Urick et al., 2016). It has received scant attention in the earlier literature on knowledge transfer between individuals while being a particularly salient feature of KTOYE. Notably, the usefulness of rapport management theory (RMT; Spencer-Oatey, 2000) in tackling interpersonal disharmony in certain contexts has been confirmed. Supporting this view, we introduced RMT into the KTOYE area and adopted a standpoint of younger employees constructing a rapport with the old for the KTOYE featured less disharmony.

Based on RMT, the increase in rapport (i.e., the harmony and smoothness in interpersonal relationships; Spencer-Oatey, 2005) is considered to be accompanied by a decrease in disharmony. As such, we disembarked the concept of rapport to explore its prominent dimensions which may play an important part in KTOYE. In addition, we highlighted the role of supportive climate (SC) as a moderator in the link between rapport dimensions (RD) and KTOYE. Using data from 318 participants of various types of companies, we found that RD structures the concept of rapport well and functions as strong catalysts in contributing to successful KTOYE. Empirical results also revealed that the degree of SC impacts the links between RD and KTOYE, meaning that the higher the level of SC, the stronger or weaker the relationships between RD and KTOYE.

Overall, three main contributions are made in this research. First, we extended the rapport literature by introducing RMT into the context of KTOYE, which broadens the theoretical scope of the distinct effects of the concept of rapport. Specifically, extricating five prominent rapport dimensions and linking them with KTOYE contributes to a revised rapport model for understanding the relational mechanism of KTOYE. Second, we advanced the literature on knowledge transfer by addressing the theoretical ambiguity in the characteristic of KTOYE (i.e., disharmony between older and younger employees) with the aid of our revised rapport model. It, therefore, initiates a plausible explanation for difficulties in KTOYE from the rapport management perspective. Third, we propose the management strategy centering on implementing rapport-building and a supportive climate is applicable across varieties of corporates that pursue smooth KTOYE. In fact, it helps firms to realize the potential of KTOYE featured by harmonious relational mechanisms as a powerful approach to the loss of organizational knowledge in the aging workplace.

This study is organized as follows: The next section presents the theoretical background and hypotheses. Subsequently, the research materials and methods are presented with the aid of quantitative data. Following that, data analysis and results are described. Thereafter, a crucial discussion and conclusion of the results are provided.

## Theoretical background and hypotheses

### Rapport management theory

Rapport management theory was first proposed by Spencer-Oatey (2000) to identify factors that influence people's dynamic perceptions of rapport when interacting or communicating with others. It has been further developed in two main areas, namely, selling and leadership. In the literature on selling, rapport makes clients feel less embarrassed, giving birth to their enjoyable interactional and verbal communication with salespeople (Campbell et al., 2006; d'Abreu et al., 2021). In leadership research, managing rapport between leaders and subordinates is a crucial driver of their high-quality relations, which subsequently increases communication satisfaction (Campbell et al., 2003; White et al., 2012). It can be seen that the role of rapport is contextually based and varies according to contextual variables such as the type of communicative activity and the nature of the communicative setting (Spencer-Oatey, 2005). As a unique communicative activity, KTOYE is a process in which participants purposefully interact and communicate with each other and become more aware of their valuable knowledge, whose effectiveness accordingly depends on the extent of rapport between participants.

In focusing on the KTOYE field, the pronounced age difference between older and younger employees leads to their dissimilar perceptions in aspects of thinking, attitude, behavior, and value systems (Bencsik et al., 2016), further characterizing KTOYE with relational disharmony. Accumulating less knowledge than their older colleagues, the younger workers give priority to receiving knowledge to be more competent. Despite possessing more knowledge about new technology sometimes (Gerpott et al., 2017), they still lack knowledge and expertise at the core of the organizations, which requires accumulating over time. In contrast, older employees have accumulated such knowledge over time (e.g., subject matter expertise, knowledge of business relationships, knowledge of governance, and knowledge of business processes; Joe et al., 2013) and are willing to share it with more efficient younger ones in the KTOYE process. For example, considering the Motomachi Plant of Toyota, where older workers impart their unique knowledge of assembling parts to younger ones who are engaged in production enables the young to learn by doing, and their productivity increases by a large margin accordingly. Nevertheless, the feature of KTOYE (i.e., the relational disharmony) makes it hard for the young to understand the expression and logic of older ones (DeLong and Storey, 2004). To cope with this, they desire relational harmony to get across and receive older colleagues' knowledge successfully. As described, RMT must be introduced into the field of KTOYE for rapport construction between younger and older employees.

In RMT, sociality rights, face, and interactional goals are suggested to structure rapport. First, sociality rights hinge upon conceptualizations of roles and fundamental principles such as equity and association. As for the former, we focused on the younger employee whose role is conceptualized as the one behaving proactively to seek knowledge (Peng et al., 2020). Considering the latter, the indifference of younger employees to authority and hierarchy makes them lay more emphasis on interactional justice (Rupčić, 2018), which presents the principle of equity. As the reflection of another principle, interactional association resonates with the needs of younger ones to supplement the lack of interpersonal bonds for needed knowledge (Ding et al., 2017). Thus, sociality rights emerged as proactive behavior (PB), interactional justice (IJ), and interactional association (IA). Second, we modified face into fear of losing face (FLF) as the young often feel afraid to be evaluated unfavorably by older counterparts (North and Fiske, 2012). Third, the younger cohort is motivated most by pushing, advancing, and reaching goals for personal success (Bencsik et al., 2016). The interactional goal is subsequently adjusted into perceived goal attainment (PGA) to measure the extent of their perceptions of being able to achieve personal goals. This study extricates the concept of rapport into five dimensions, namely, IJ, PB, FLF, PGA, and IA.

### Knowledge transfer from older to younger employees

Knowledge transfer from older to younger employees is important as it retains the valuable knowledge of older workers to nurture more knowledgeable younger workers for the maintenance of competitive advantages of firms. Given that knowledge of the older employees is mainly tacit, it requires positive personal relationships to be transferred (Martins and Meyer, 2012; Rooney et al., 2013).

Investigations into trust and willingness have enjoyed the greatest popularity in the existing literature on relationships between knowledge sender and receiver. In the KTOYE context, older employees' future time at work is limited, making them willing to pass on their knowledge to fulfill the requirements of guiding younger colleagues (Doerwald et al., 2021; Fasbender and Gerpott, 2021). By comparison, younger ones have accumulated far less knowledge, thus expressing more willingness to receive knowledge from older employees to satisfy achievement needs at work (Kooij et al., 2011). Concerning trust, older employees are deemed as someone trustworthy by their younger counterparts because of their knowledge developed over years (Wikström et al., 2018). Correspondingly, the propensity of younger employees to invest mental effort in knowledge acquisition may touch older ones and earn their trust (Fasbender et al., 2021). However, most of these studies have ignored the age difference between the two cohorts in generating their dissimilar perceptions in aspects of thinking, behavior, value systems, etc. (Schmidt and Muehlfeld, 2017), which leads to relational disharmony in KTOYE. Despite a high level of trust and willingness to share knowledge demonstrated by older workers, their knowledge still suffers from the relational disharmony to flow freely to younger employees. Particularly, it was documented that younger workers desire harmony in relationships with the old for smooth communications (Zhang et al., 2005). Thus, our research strives to unpack the concept of rapport in RMT and explore how its prominent dimensions function to promote the successful KTOYE from the perspective of younger employees.

### Interactional justice and KTOYE

Interactional justice is defined as the perceived fairness of employees based on whether they are treated by an authority figure with dignity, personal care, respect, and trust (Colquitt, 2001; Gupta et al., 2021). Given that younger employees attach less importance to authority and hierarchy, older ones who treat them fairly are the ones with whom they prefer to communicate (Rupčić, 2018). In addition, getting equal treatment from older colleagues could make them feel comfortable and enjoyable, propelling the formation of psychological bonds between the two cohorts (Hyun and Kim, 2014). This positive psychological state thereupon strengthens the affective commitment (Thompson and Heron, 2005; López-Cabarcos et al., 2016) of younger ones to further take part in receiving knowledge from the old. Furthermore, IJ appears relevant to the feelings of younger employees about being accepted and included within a group, which strengthens their sense of self-worth (Xiang et al., 2019) and prompts them to acquire knowledge from the old.

**H1**. IJ has a positive impact on KTOYE.

### Proactive behavior and KTOYE

Proactive behavior refers to “taking initiative in improving current circumstances; it involves challenging the status quo rather than passively adapting present conditions” (Crant, 2000). Proactive employees tend to behave with some degree of enactive mastery and controllability of a situation (Parker et al., 2006). As such, often being newcomers to organizations, younger employees place a much higher emphasis on proactive behavior in the process of KTOYE through which they gain more knowledge for a remedy for psychological uncertainty (Li et al., 2011; Peng et al., 2020). It has been previously observed that the knowledge-based behaviors of recipients form the beliefs and attitudes of sharers, enabling the sharer to behave accordingly (Lichtenstein and Hunter, 2006). PB signifies that younger workers accord particular importance to the knowledge of older employees, making the old feel confident since their knowledge and skills are valued (Fasbender and Gerpott, 2021), and in turn ardently share more knowledge with the young.

**H2**. PB has a positive impact on KTOYE.

### Fear of losing face and KTOYE

Fear of losing face is, at its core, a feeling that relies heavily on the importance of preventing undesirable events such as being devalued or even stigmatized (Kim and Yon, 2019; Zhao and Zhu, 2021). If employees' exposure to failure experiences or knowledge is considered useless, their feelings of embarrassment, shame, or dishonor may be evoked (Zhang and Ng, 2012). In particular, younger ones are short of well-developed knowledge, which blocks their feelings of self-confidence (Kim and Ok, 2010) and increases the possibility of facing threats. Concerns about face loss indicate their worry that older employees may evaluate them unfavorably, further restricting their active participation in KTOYE (Fasbender and Gerpott, 2021). In addition, the fact that most of them are of relatively lower status hardly protects them against the concern of face loss (Fasbender and Gerpott, 2021). As a consequence, the younger cohort is liable to be quieter in KTOYE, namely, they may ask fewer questions. It subsequently impedes them to receive knowledge from older ones (Gerpott and Fasbender, 2020).

**H3**. FLF has a negative impact on KTOYE.

### Perceived goal attainment and KTOYE

Driven by the need for the achievement of instrumental or knowledge-related goals, younger workers tend to be actively involved in KTOYE (Burmeister et al., 2020). Swift et al. (2010) identified two main types of goal orientations, namely, learning and performance goal orientations. PGA of younger employees with two orientations serves as the lubricant for KTOYE. Those who are driven by a learning goal tend to acquire new knowledge and improve capabilities, competence, and mastery (Swift et al., 2010; Shariq et al., 2019) through participating in KTOYE. On recognizing a high degree of learning goal attainment, younger workers may get more engaged in KTOYE as they want to perform better compared to their previous performance (Kim and Lee, 2013). For those with a performance goal orientation who desire to outperform other youngsters, it is crucial to acquire knowledge and especially positive evaluations from the old, which greatly enhance their self-image (Yun et al., 2007). Performance goals perceived to be attained with a high probability could promote younger ones to take part in KTOYE.

**H4**. PGA has a positive impact on KTOYE.

### Interactional association and KTOYE

Younger employees being in pursuit of a feeling of association with the old are motivated to engage in the process of KTOYE, for which their social relationships could be solidified (Beal et al., 2003; Burmeister et al., 2020). IA means that people have an entitlement to develop relationships with others for social involvement (Spencer-Oatey, 2005). It is established based on perceived similarities predicting the strength of mutual understanding, care, and trust (Pillemer and Rothbard, 2018), which provoke the propensity of younger employees to acquire knowledge (McNichols, 2010; Martins and Meyer, 2012). For the young, it is probably the most challenging experience to confront the lack of developed interpersonal relationships that could provide them with needed knowledge and resources (Ding et al., 2017). As such, the maintenance of IA improving relationship closeness could function as a relational enhancer for younger workers' acquisition of valuable knowledge (Su et al., 2009; Wang et al., 2012).

**H5**. IA has a positive impact on KTOYE.

### The moderation effect of the supportive climate

As proposed by Wang et al. (2017), the supportive climate represents a certain KTOYE climate where “older and younger employees can trust each other; can collaborate, openly and honestly communicate with, and build a friendly relationship with each other; and can be treated equitably in the organization”. The findings of their research showed that SC has a direct positive influence on KTOYE. When the organizational climate is supportive, younger workers may perceive the workplace as cohesive and inclusive and then take a more active part in knowledge-collecting behaviors (Lagacé et al., 2019). In addition, SC makes younger employees feel empowered to learn and get actively involved in the transfer of knowledge (Uhunoma et al., 2021).

**H6**. SC has a positive impact on KTOYE.

It has been suggested that the role of rapport not only varies according to the type of communicative activity (i.e., KTOYE studied in this research) but also hinges upon the nature of the communicative setting (Spencer-Oatey, 2005). Notably, the organizational climate can be a moderator in influencing the relationship between knowledge transfer and its antecedents (Van Wijk et al., 2008). We identify the organizational level SC as a type of communicative setting that moderates the links between rapport dimensions and KTOYE. First, perceptions of younger employees of IJ could be greatly increased to promote KTOYE when the organizational climate is supportive and employees of all backgrounds are understood and treated with respect and honesty (Colquitt, 2001; Nishii, 2012). In addition, SC prompts open and honest communication, through which older employees provide younger employees with clear explanations for workplace changes. Younger ones may subsequently feel that IJ is enhanced (Kernan and Hanges, 2002), thereby being encouraged to participate in the process of KTOYE. Second, the higher level of SC the young perceive, the more proactive behavior they will display to acquire knowledge from older ones. Two factors could account for it. On one hand, younger employees have more confidence in their abilities within SC since they are trusted by older coworkers who accept their mistakes as learning experiences, enabling them to proactively try things beyond core tasks (Parker et al., 2006; Hong et al., 2016). On the other hand, SC provides more developmental feedback to the younger cohort (Boehm and Dwertmann, 2014), further contributing to the proactivity of their behavioral pattern (Li et al., 2011). Based on these, younger employees will have more cravings for KTOYE. Third, working in the SC, they concern less about face loss and engage more actively in KTOYE. This is because SC makes them freely voice their opinions without the fear of being subject to feelings of ignominy for their mistakes and incompetence (Magni et al., 2018; Wolfson et al., 2018). Moreover, SC supports collaboration and restrains informal competitiveness, revealing that the climate opposes the gain of one person against others (Gerpott and Fasbender, 2020). Under this circumstance, the competitiveness of younger employees is less likely to be regarded as a threat to older ones. It leads to less conflicting situations where the young could be exempt from losing face (Orth et al., 2010; Henry et al., 2018) and therefore seek more knowledge from older workers. Fourth, when the climate is supportive, younger employees could openly communicate with the old and thus have more chances to reveal their true selves in the KTOYE process (Roberson and Block, 2001). It increases the accuracy of their goals to be understood and supported and contributes to the higher degree of their PGA conducive to the continuous KTOYE. Besides, younger and older cohorts working under SC are encouraged to display supportive behaviors by looking out for their interests and goals in addition to their own (Beersma et al., 2003; Johnson et al., 2006; Cerne et al., 2013). That increases the possibility that goals of both younger and older workers could be attained and facilitates the KTOYE that may benefit further goal attainment. Fifth, in an organization highlighting SC, IA between younger and older workers is more intimate, which brings the smoothness of KTOYE to a larger extent. Shared cognition between two cohorts enhanced by SC accounts in part for this. To clarify exactly, SC makes younger employees interact and communicate more often with the old, thus shaping shared cognition between them (Newell et al., 2004; Lefebvre et al., 2016). The shared cognition gives birth to IA with more interpersonal attraction and mutual intimacy (Li et al., 2013), which enables the young to obtain unsolicited knowledge of older workers. Apart from it, the development of intergenerational trust driven by SC plays another key role in animating employees to increase investment in IA (Parzefall and Kuppelwieser, 2012), further opening doors to social networks that offer the young broader access to needed knowledge (Murray and Fu, 2016).

**H6a**. SC positively moderates the relationship between IJ and KTOYE.
**H6b**. SC positively moderates the relationship between PB and KTOYE.
**H6c**. SC positively moderates the relationship between FLF and KTOYE.
**H6d**. SC positively moderates the relationship between PGA and KTOYE.
**H6e**. SC positively moderates the relationship between IA and KTOYE.

**Figure 1:**
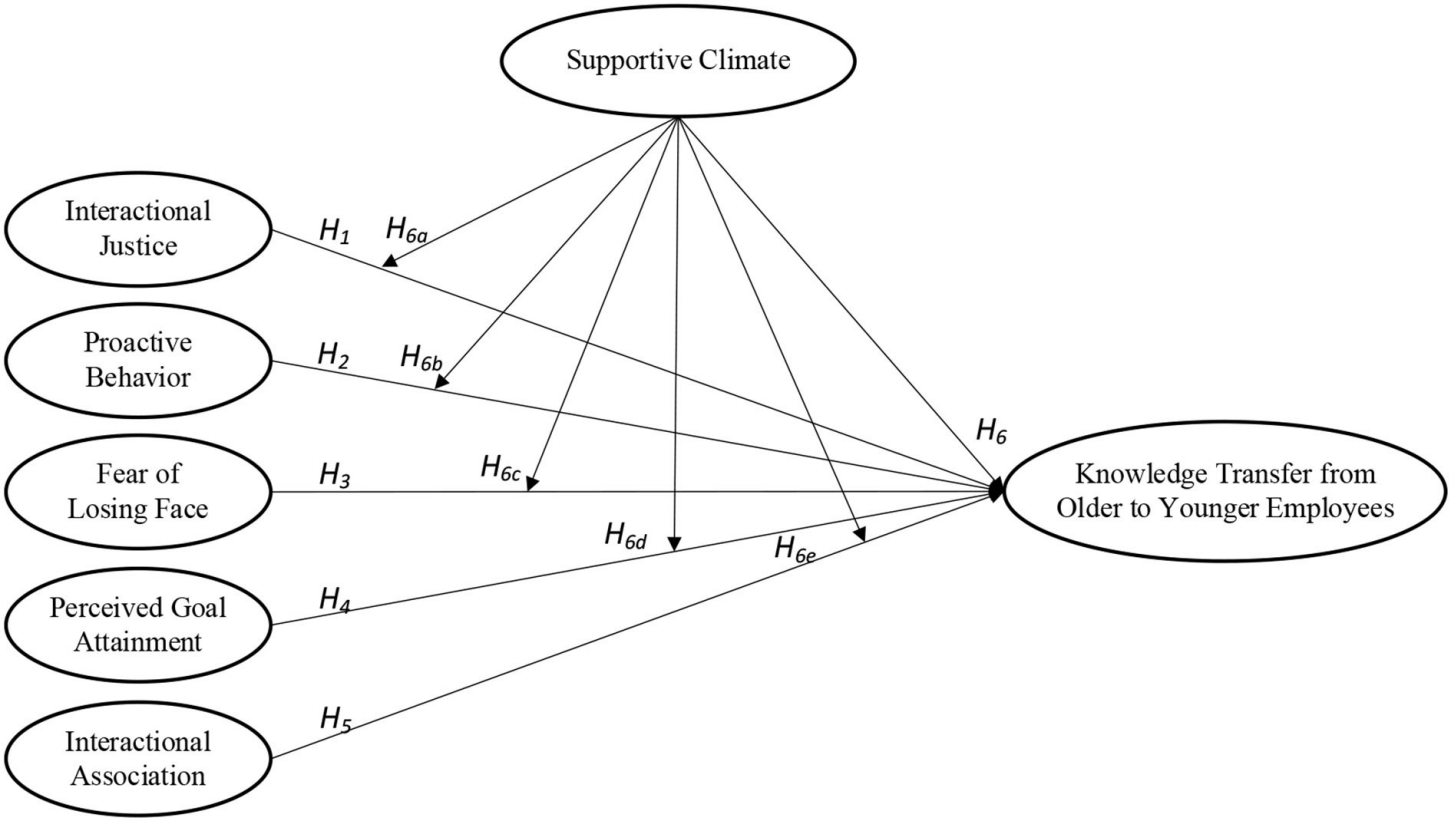
Conceptual model.

Based on the above theoretical groundwork, the conceptual model was built as shown in Figure 1.

## Materials and methods

### Sample and data collection

To test the research hypotheses, quantitative data were collected from a sample of younger employees aged 40 years or below from different industries across China. Our decision to reach these participants was driven by the intention to arrive at generalized conclusions applicable to various corporates. We sent the online link of the questionnaire to corporates and informed respondents of the anonymity and confidentiality of their responses in advance. In doing so, 439 questionnaires were received in total. After excluding invalid questionnaires, 318 responses were selected for the final sample, resulting in a satisfying response rate of 72.4%.

The main characteristics of survey respondents are summarized in Table 1. Of the participants, 51.9% are women and 48.1% are men. The majority of them are aged 35 years or below (79.9%), and most have been with their companies for 10 years or below (61.3%), which could be a good proxy for younger workers in China. Moreover, they come from a wide variety of industries, incorporating the following: financial (19.5%), IT (18.2%), manufacturing (16.7%), energy and mining (12%), construction (7.9%), culture (4.7%), education (4.1%), retail (4.1%), logistics (3.1%), telecommunication (3.1%), healthcare (2.5%), real estate (2.5%), and consulting (1.6%).

**Table 1:** Sample characteristics.

**Sample characteristic**	**Items**	**Frequency**	**(%)**
Age	Under 30 years old	121	38.1
	30–35 years old	133	41.8
	36–40 years old	64	20.1
Gender	Female	165	51.9
	Male	153	48.1
Tenure	Under 5 years old	91	28.6
	5–10 years old	104	32.7
	Over 10 years old	123	38.7
Industry type	Financial	62	19.5
	IT	58	18.2
	Manufacturing	53	16.7
	Energy and mining	38	12
	Construction	25	7.9
	Culture	15	4.7
	Education	13	4.1
	Retail	13	4.1
	Logistics	10	3.1
	Telecommunication	10	3.1
	Healthcare	8	2.5
	Real estate	8	2.5
	Consulting	5	1.6

### Selection of variables and scales

The conceptual model consists of seven variables that were measured using multiple-item scales adopted from previous studies. All the items assessed by respondents were rated on a five-point Likert-type scale (ranging from 1 = strongly agree to 5 = strongly disagree).

#### Knowledge transfer from older to younger employees

It was accessed using five items adapted from the study of Wang et al. (2017). An example item is “I can acquire key ideas, concepts, or theories in the field of expertise from older employees.”

#### Interactional justice

It was assessed using four items from the scale of Niehoff and Moorman (1993) and one item adopted from the scale of Rupp and Cropanzano (2002). An example item is “When transferring knowledge with older employees, they treat me with respect and dignity.”

#### Proactive behavior

It was measured using three items from Belschak and Den Hartog's (2010) scale. An example item is “I take the initiative to help older employees with developing or implementing new ideas.”

#### Fear of losing face

It was measured using the scale of Zane and Yeh (2002). The example item is “During a discussion, I try not to ask questions because I may appear ignorant to older employees.”

#### Perceived goal attainment

It was assessed using five items adapted from the scale of Button et al. (1996). The example item is “When transferring knowledge with older employees, I have the opportunity to learn new things.”

#### Interactional association

It was measured using Bock et al.'s (2005) measure for the anticipated reciprocal relationships. The example item is “Older employees' sharing of knowledge with me would strengthen our ties.”

#### Supportive climate

It was measured using five items from the scale of Wang et al. (2017). The example item is “In our organization, employees of different ages can get along well with each other.”

#### Control variables

We controlled for the age, gender, and tenure of participants, which were frequently used to test individual-level hypotheses in knowledge transfer studies (Wang et al., 2017; Burmeister et al., 2020).

### Reliability and validity of the scale

To test the reliability of the measures, we examined the respective Cronbach's alpha coefficients of seven variables, which are all greater than the recommended level of 0.7. To validate the measurement, the degree of fit of the model, convergent validity, and discriminant validity were evaluated.

We first assessed the following indicators: absolute fit measures, including chi-square/df (CMIN/df), the goodness of fit index (GFI), standardized root-mean-square residual (SRMR), and root-mean-square error of approximation (RMSEA); relative fit measures, including Tucker-Lewis index (TLI), comparative fit index (CFI), and incremental fit index (IFI). As reported in Table 2, all fit indices achieve satisfactory levels.

**Table 2:** Model fit indices.

**Fit indices**	**Scores**	**Recommended**
**Absolute indices**		
CMIN/df	2.060	<5
GFI	0.855	>0.8
SRMR	0.043	<0.06
RMSEA	0.058	<0.08
**Relative indices**		
TLI	0.918	>0.9
CFI	0.927	>0.9
IFI	0.928	>0.9

According to Hair et al. (2010), several ways are available to evaluate the convergent validity: (1) standardized loading estimates of 0.5 or greater and 0.7 or higher is ideal; (2) an average variance extracted (AVE) of 0.5 or higher suggests adequate convergence; and (3) a composite reliability (CR) value of 0.7 or higher is also an indicator of good convergent validity. As shown in Table 3, all factor loadings range from 0.608 to 0.868 (all >0.6), being significant at the level of 0.001. AVE values ranging from 0.545 to 0.652 are higher than 0.5, and the CR values range from 0.791 to 0.903 (all >0.7). Consequently, the measurement suggests adequate convergent validity.

**Table 3:** Validity and reliability of the measurement model.

**Factors**	**Loading**	**AVE**	**CR**	**Cα**
**Interactional justice (IJ)**				
When transferring knowledge with older employees				
IJ1. They treat me with respect and dignity	0.682^***^	0.545	0.857	0.856
IJ2. They deal with me in a truthful manner	0.746^***^			
IJ3. They offer adequate justification for decisions made about my job	0.761^***^			
IJ4. Their decisions are made out in the open so that everyone always knows what's going on	0.755^***^			
IJ5. They explain very clearly any decision made about my job	0.744^***^			
**Proactive behavior (PB)**				
PB1. I take the initiative to take over older employees' tasks when needed even though I am not obliged to	0.815^***^	0.559	0.791	0.787
PB2. I take the initiative to help older employees with developing or implementing new ideas	0.728^***^			
PB3. I take the initiative to take on tasks that will further my career	0.695^***^			
**Fear of losing face (FLF)**				
FLF1. During a discussion, I try not to ask questions because I may appear ignorant to older employees	0.834^***^	0.547	0.827	0.825
FLF2. I maintain a low profile because I do not want to make mistakes in front of older employees	0.794^***^			
FLF3. I downplay my abilities and achievements so that older employees do not have unrealistically high expectations of me	0.663^***^			
FLF4. When an older employee criticizes me, I try to avoid him/her	0.651^***^			
**Perceived goal attainment (PGA)**				
When transferring knowledge with older employees				
PGA1. I have the opportunity to learn new things	0.807^***^	0.652	0.903	0.901
PGA2. I have the opportunity to extend the range of my abilities	0.876^***^			
PGA3. I have the opportunity to do challenging work	0.796^***^			
PGA4. I have the opportunity to improve on my past performance	0.797^***^			
PGA5. I have the opportunity to impress them by doing a good job	0.755^***^			
**Interactional association (IA)**				
IA1. Older employees' sharing of knowledge with me would strengthen our ties	0.768^***^	0.589	0.851	0.851
IA2. Older employees' sharing of knowledge with me would expand the scope of my association with others in the organization	0.774^***^			
IA3. Older employees' sharing of knowledge with me would smooth our cooperation in the future	0.793^***^			
IA4. Older employees' sharing of knowledge with me would help me create strong relationships with those who have common interests as me in the organization	0.733^***^			
**Supportive climate (SC)**				
SC1. In our organization, employees of different ages can trust each other	0.786^***^	0.582	0.873	0.862
SC2. Our organization can treat employees of different ages equitably in staff training, performance appraisal, pay systems, etc.	0.608^***^			
SC3. In our organization, employees of different ages can get along well with each other	0.788^***^			
SC4. In our organization, employees of different ages can speak freely to each other	0.739^***^			
SC5. In our organization, employees of different ages can build a good intergenerational relationship	0.868^***^			
**Knowledge transfer from older to younger employees (KTOYE)**				
KTOYE1. I can acquire key ideas, concepts or theories in the field of expertise from older employees	0.813^***^	0.637	0.898	0.897
KTOYE2. I can learn about recent advances in the field of expertise from older employees	0.801^***^			
KTOYE3. I can acquire experience or know-how from older employees	0.819^***^			
KTOYE4. I can acquire best practices or ways to solve problems from older employees	0.754^***^			
KTOYE5. I can acquire tips on jobs from older employees	0.803^***^			

N = 318;

*** p < 0.001.

Discriminant validity is the extent to which a construct is truly shown to be discriminable from other constructs. A rigorous way to test it is to compare the square root of the AVE value of each construct with the correlation coefficients between it and any other construct. The square root of the AVE value should be greater than the correlation coefficients. Table 4 illustrates that the square root of the AVE value for each construct is higher than the correlation coefficients in the same row and column, demonstrating good discriminant validity. Given the above, the results could be supportive evidence for the satisfactory model fit, reliability, and validity of the scale.

**Table 4:** Descriptive statistics and correlation matrix.

**Construct**	**Mean**	**SD**	**IJ**	**PB**	**FLF**	**PGA**	**IA**	**SC**	**KTOYE**
IJ	2.403	0.687	**0.738**						
PB	2.068	0.624	0.395^**^	**0.748**					
FLF	3.262	0.850	0.068	0.106	**0.740**				
PGA	1.942	0.598	0.441^**^	0.468^**^	−0.028	**0.807**			
IA	2.045	0.601	0.508^**^	0.495^**^	0.061	0.585^**^	**0.767**		
SC	2.346	0.689	0.572^**^	0.412^**^	0.047	0.432^**^	0.495^**^	**0.763**	
KTOYE	2.066	0.596	0.468^**^	0.525^**^	0.076	0.637^**^	0.592^**^	0.543^**^	**0.798**

** p < 0.01.

The results marked in bold indicate the square root of AVE (diagonal elements) for each construct, and results off the diagonal represent the correlation coefficients for each construct in the relevant rows and columns.

## Data analysis and results

To test all hypotheses, we analyzed the data using hierarchical multiple regression. IJ, PB, FLF, PGA, IA, and SC were standardized before their interaction terms were calculated to avoid the problem of multicollinearity. As shown in Table 5, Model 1 was created to test the effects of control variables (age, gender, and tenure) on the dependent variable (KTOYE). In Model 2, the direct impacts of independent variables (IJ, PB, FLF, PGA, and IA) on KTOYE were assessed. Then, Model 3 was built to examine the influences of independent variables and the moderator (SC) on KTOYE. Ultimately, in Model 4, the moderating effects of SC on the relationship between independent variables and KTOYE were presented. To examine moderating effects, we generated interaction terms by multiplying the independent variables with the moderator.

**Table 5:** Results of the hierarchical linear regression analysis.

**Dependent variable:** **Knowledge transfer from older to younger employees**	**Model 1**		**Model 2**		**Model 3**		**Model 4**	
**Control variables**								
Age	−0.010	(−0.092)	0.042	(0.534)	0.064	(0.837)	0.052	(0.695)
Gender	0.008	(0.067)	−0.053	(−0.672)	−0.036	(−0.460)	−0.054	(−0.708)
Tenure	−0.009	(−0.084)	−0.059	(−0.824)	−0.061	(−0.875)	−0.047	(−0.682)
**Focus variables**								
Interactional justice(A)			0.118^*^	(2.490)	0.033	(0.666)	0.076	(1.460)
Proactive behavior(B)			0.192^***^	(4.008)	0.167^***^	(3.564)	0.143^**^	(3.015)
Fear of losing face(C)			0.043	(1.085)	0.045	(1.150)	0.042	(1.032)
Perceived goal attainment(D)			0.367^***^	(7.160)	0.345^***^	(6.898)	0.368^***^	(7.297)
Interactional association(E)			0.224^***^	(4.182)	0.185^***^	(3.499)	0.132^*^	(2.399)
Supportive climate(F)					0.215^***^	(4.323)	0.234^***^	(4.718)
**Interactions**								
Interaction A × F							−0.097^*^	(−2.334)
Interaction B × F							0.005	(0.123)
Interaction C × F							0.040	(1.102)
Interaction D × F							−0.108^*^	(−2.391)
Interaction E × F							0.125^**^	(2.676)
R^2^	0.000		0.525		0.553		0.579	
Adjusted R^2^	−0.009		0.513		0.540		0.558	
F-value	0.022		42.758^***^		42.258^***^		29.775^***^	

* p < 0.05;

** p < 0.01;

*** p < 0.001;

t-values in parentheses.

In the first step (Model 1), the direct effects of control variables (age, gender, and tenure) on KTOYE were examined. Yet, no control variables were found to have a significant effect on KTOYE.

In the second step (Model 2), IJ significantly enhanced KTOYE (β = 0.118, t = 2.490), supporting H_1_. As expected, PB was positively related to KTOYE (β = 0.192, t = 4.008), supporting H_2_. However, not as we predicted, FLF had no significant effect on KTOYE (β = 0.043, t = 1.085). Thus, H_3_ is rejected. The results also reveal that PGA positively correlates with KTOYE (β = 0.367, t = 7.160). Thus, H_4_ is supported. Besides, it can be seen that IA has a significant positive impact on KTOYE (β = 0.224, t = 4.182), supporting H_5_.

In the third step (Model 3), consistent with our expectation, SC positively affects KTOYE (β = 0.215, t = 4.323). Thus, H_6_ is supported. Interestingly, IJ was observed to have a nonsignificant impact on KTOYE (β = 0.033, t = 0.666) when SC was added into the model, which therefore contradicts H_1_ suggesting that IJ is positively related to KTOYE.

In the last step (Model 4), contrary to H_6a_, the impact of SC on the relationship between IJ and SC is significant but negative (β = −0.097, t = −2.334). In addition, the results suggest a nonsignificant effect of SC on the relationship between PB and KTOYE (β = 0.005, t = 0.123). Hence, H_6b_ is rejected. SC has a nonsignificant moderating effect on the relationship between FLF and KTOYE (β = 0.040, t = 1.102), rejecting H_6c_. The effect of SC on the relationship between PGA and KTOYE is significant but negative (β = −0.108, t = −2.391), thus not supporting H_6d_. As we hypothesized, SC significantly enhances the relationship between IA and KTOYE (β = 0.125, t = 2.676), supporting H_6._

## Discussion and conclusion

The aging and shrinking of knowledgeable older workers entail the organization to transfer their important knowledge to younger ones. Considering the characteristic of KTOYE, rapport plays a particularly crucial role to promote successful KTOYE. Thus, we drew RMT for quantitative analysis of five RD, namely, IJ, PB, FLF, PGA, and IA, as well as SC in KTOYE of impacting firms. Our results demonstrate that FLF, IJ, PB, PGA, IA, and SC have different impacts on KTOYE: (1) FLF was found not to prompt younger workers to engage in KTOYE. A possible explanation is that younger ones believe losing face in front of clients is something worse and therefore have a higher tolerance for face threats on the condition that they can obtain knowledge from older workers to serve clients better (Ardichvili et al., 2006); (2) we found that IJ significantly contributes to KTOYE, indicating that KTOYE with the low level of IJ will be plagued by the relational disharmony that hinders knowledge acquisition of younger ones. IJ enables the young to be treated with respect, trust, and personal care, further making them enjoyable to receive knowledge from the old (Yeşil and Dereli, 2013; Phong and Son, 2020); (3) PB's positive impact on KTOYE was found to reveal that the more proactively the younger workers behave, the more knowledge they will obtain from the old. This is because their proactivity could give older ones the recognition that drives them to share more knowledge (Fasbender and Gerpott, 2021); (4) a positive link between PGA and KTOYE was discovered here, implying that the high degree of PGA inspires younger ones to engage in KTOYE. The high probability to gain capabilities, competence, and mastery predicted by high-level PGA could account for the active participation of younger ones in KTOYE (Swift et al., 2010; Shariq et al., 2019); (5) IA was found to enhance KTOYE since it facilitates the formation of perceived similarity between younger and older workers, which acts as a conduit for KTOYE (McNichols, 2010; Martins and Meyer, 2012); and (6) as predicted, SC was found to be an important facilitator in the process of KTOYE. It results from the fact that when the climate is perceived to be supportive, younger ones may feel empowered to display active knowledge-collecting behaviors (Lagacé et al., 2019).

**Figure 2:**
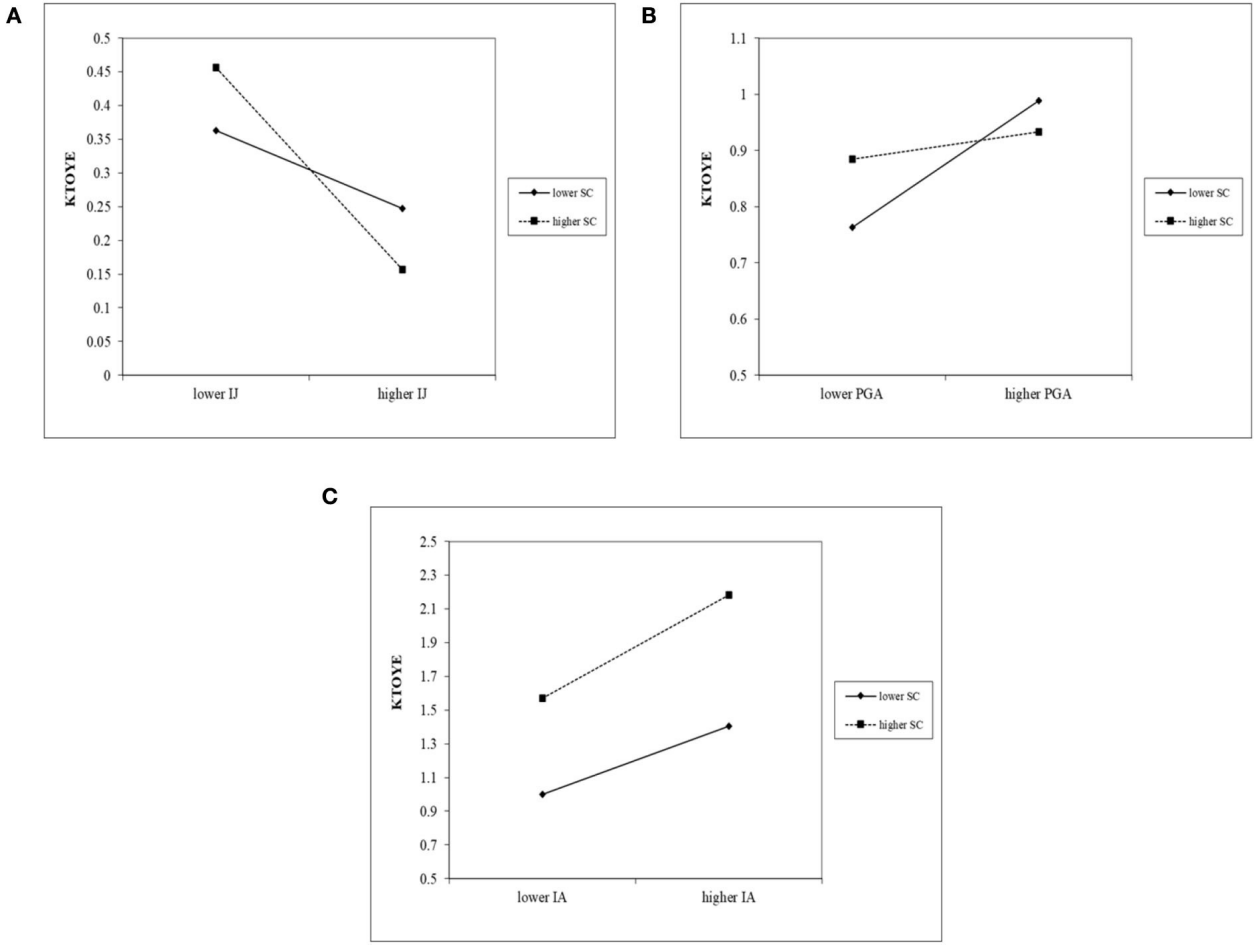
Plots of moderating effects. **(A)** Moderating effect of SC on the relationship between IJ and KTOYE; **(B)** Moderating effect of SC on the relationship between PGA and KTOYE; **(C)** Moderating effect of SC on the relationship between IA and KTOYE; IJ, interactional justice; PGA, perceived goal attainment; IA, interactional association; SC, supportive climate; KTOYE, knowledge transfer from older to younger employees.

Furthermore, the moderating effect of SC is strong that it enhances the positive influence of IA on KTOYE and even replaces the importance of IJ and PGA on KTOYE: (1) SC was found to positively moderate the link between IA and KTOYE as it strengthens the shared cognition between younger and older cohorts, which increases the quality of IA and thus propels the transfer of their knowledge (Parzefall and Kuppelwieser, 2012; Lefebvre et al., 2016); (2) although IJ and SC facilitate KTOYE, respectively, their interaction negatively impacts KTOYE (Figure 2). In addition, the existence of SC leads to a nonsignificant positive effect of IJ on KTOYE (Table 5), implying that the importance of IJ is fully replaced by SC. This is probably because SC enables younger and older employees to obtain various organizational justice (i.e., distributive, procedural, interactional, and informational justice), which are proven to boost KTOYE (Schmitt et al., 2012). Consequently, SC contributes to the increase in a broader range of organizational justice and exerts more significant positive influences on KTOYE than IJ, further playing a negative role in moderating the relationship between IJ and KTOYE; and (3) the negative moderation effect of SC on the relationship between PGA and KTOYE exists, but both PGA and SC originally serve as motivators to KTOYE. PGA still significantly enhances KTOYE when being accompanied by SC (Table 5), meaning that SC replaces the partial importance of PGA on IGKT. This result derives from the fact that SC promotes the establishment of shared goals between older and younger workers (Samadi et al., 2015), which have a greater probability to be achieved than personal goals in the collaborative KTOYE process. Compared with PGA focusing more on personal goals, the attainment of common goals could be a more essential precursor to KTOYE. Thus, SC lessens the importance of PGA and negatively moderates its relationship with KTOYE.

### Theoretical implications

This research makes several theoretical contributions to existing literature. First, we extend rapport research by using RMT as the theoretical foundation of links between RD and KTOYE. Previous studies mostly investigate selling, service, and interviewing contexts (Kim and Ok, 2010; Jenner and Myers, 2019; d'Abreu et al., 2021) and seldom pay attention to specifying the concept of rapport. First introducing rapport into the KTOYE context, this study structures rapport with five critical dimensions based on RMT and the characteristic of KTOYE. Our results evidence the significantly positive influence of almost all rapport dimensions on KTOYE, revealing that rapport can be well structured across these key dimensions, and function adequately in the process of KTOYE. Thus, this study offers further insights into the contextually based rapport and a revised model of rapport in enriching the relational mechanism of KTOYE, which contributes to expanding the theoretical scope of RMT correspondingly.

Second, this study advances the knowledge transfer literature by clarifying and overcoming the characteristic of KTOYE (i.e., disharmony between older and younger employees). Given the workplace aging and youth-centeredness, KTOYE becomes an increasingly important part of knowledge transfer literature. We argue that trust and willingness (Anand et al., 2019; Bacon et al., 2020; Nguyen et al., 2020) seem not to be the main influencing factors in the context of KTOYE. Even with the high level of trust and willingness, the KTOYE process remains difficult. It is the ambiguity in the characteristics of KTOYE that accounts for such difficulty, which has still received scant attention in the research literature. Given this, we confirm that our reformulation of the rapport model contributes to providing a powerful explanation of how the smoothness of KTOYE is brought about by its harmonious relational mechanism.

Third, this study deepens the understanding of the moderating role of SC in the relationship between RD and KTOYE. We find that SC determines the extent to which younger employees have a rapport with older colleagues to acquire knowledge effectively. Simply put, there is theoretical evidence of the moderation effect of SC in a harmony-based relational mechanism of KTOYE. When SC is strong, firms can spend less effort cultivating rapport between older and younger employees for the success of KTOYE. This advances previous studies merely on the direct effect of SC on intergenerational knowledge transfer (McNichols, 2010; Wang et al., 2017). Thereby, taking SC as a moderator specifies the boundary conditions for the effects of rapport on the general KTOYE characterized by relational disharmony.

### Practical implications

This study provides several practical guidance for managers. First, managers should provide ample opportunities for younger employees to connect and collaborate with the old (e.g., teamwork or networking; McNichols, 2010; Saks et al., 2011). In this way, strong relationships between two cohorts could be fostered, through which the younger cohort could have access to the knowledge they want, as IA was proven to positively influence KTOYE. In addition, collaboration-orientated interaction between two cohorts offers more developmental feedback to younger employees and has their mistakes accepted as a learning experience more often, contributing to their proactive behaviors that play a key role in KTOYE. Our results, indicating the significant positive effect of PB on KTOYE, can support this.

Second, managers should encourage open communication between younger and older workers. This enables the young to get a clear and reasonable explanation of workplace changes from older workers and then recognize more IJ that promotes their participation in KTOYE (Kernan and Hanges, 2002). In addition, the true selves revealed in mutual communication make the goals of younger employees supported more accurately by the older cohort and get involved in KTOYE a step further (Roberson and Block, 2001) since IJ and PGA were evidenced to stimulate knowledge transfer of younger workers' with the old.

Third, managers should foster SC in organizations to provide younger workers with psychological forces to seek relational harmony that facilitates their acquisition of knowledge from the old (Uhunoma et al., 2021), especially when they suffer from low levels of IA, IJ, and PGA that undermine their intrinsic motivation to participate in KTOYE. SC was observed to positively moderate the link between IA and KTOYE, completely replace the importance of IJ on KTOYE, and partially substitute for the effect of PGA on KTOYE.

### Limitations and future research

Although this study has various strengths that advance KTOYE literature, it still suffers from a few limitations. On the one hand, a potential problem within this research is its sole focus on the direct influences of RD on the involvement of younger employees in KTOYE. The data indicate that there may be intercorrelations between the five dimensions of rapport. Hence, the attention of future research needs to be directed to the complicated relationships among IJ, PB, FLF, PGA, and IA. On the other hand, the cross-sectional design of our study has not been without criticism. Though conferring benefits for generational issues (Lyons and Kuron, 2014), it may cause the findings to be changeable. For future research, a reasonable approach to tackle this problem could be combining cross-sectional and longitudinal designs to further confirm the findings.

## Data availability statement

The raw data supporting the conclusions of this article will be made available by the authors, without undue reservation.

## Author contributions

All authors listed have made a substantial, direct, and intellectual contribution to the work and approved it for publication.
